# The Rate of Progression of Geographic Atrophy Decreases With Increasing Baseline Lesion Size Even After the Square Root Transformation

**DOI:** 10.1167/tvst.7.6.40

**Published:** 2018-12-28

**Authors:** Jordi Monés, Marc Biarnés

**Affiliations:** jmones@institutmacula.com; mbiarnes@institutmacula.com; 1 Institut de la màcula (Hospital Quirón Teknon), Barcelona, Spain; 2 Barcelona Macula Foundation, Barcelona, Spain

**Keywords:** age-related macular degeneration, geographic atrophy, progression, square root transformation

## Abstract

**Purpose:**

To determine the relationship between the progression of geographic atrophy (GA) and its baseline area (BA) using the square root transformation (sqrt) for different atrophy sizes.

**Methods:**

Single eyes of patients with GA visiting the Institut de la Màcula (Barcelona, Spain) between December 2009 and January 2018 with a follow-up of ≥6 months were included. The main outcome was the correlation between BA and growth after the sqrt using Pearson's *r* and Spearman's *rho*. The graphical relationship was explored using linear and LOWESS regression. In a secondary, prespecified analysis, progression was compared by BA categories (Age-Related Eye Disease Study [AREDS] classification and BA tertiles). In post hoc analyses, the results were evaluated in subgroups defined by location of atrophy, number of lesions, fundus autofluorescence pattern, and fellow-eye status.

**Results:**

We included 128 eyes (mean follow-up, 3.1 years). The correlation between BA and progression was negative (*r* = −0.30, *P* = 0.0005; *rho* = −0.25, *P* = 0.0042). There was a decrease in the rate of progression in mm/year with increasing BA, but this was significant for tertiles (*P* = 0.0078) and not AREDS (*P* = 0.20). The descending trend was driven by high-risk features.

**Conclusions:**

The correlation between GA progression and BA using the sqrt is negative. This has implications for the expected prediction of progression of a given lesion and to avoid overestimating the beneficial effects of interventional therapies.

**Translational Relevance:**

The GA progression/BA relationship using the sqrt currently is regarded as independent. Our results suggest the sqrt slope actually is negative, which should be kept in mind to avoid misinterpretation of results in advanced therapies.

## Introduction

Geographic atrophy (GA) secondary to age-related macular degeneration (AMD) is characterized by the progressive enlargement of atrophy of the retinal pigment epithelium (RPE) as well as photoreceptor and choriocapillaris loss.^1^ Progression of atrophy is negatively related to visual function and everyday task activities,^2–5^ and it is now accepted as an endpoint in clinical trials.^6^

Baseline area of atrophy (BA) is a predictor of progression rate over the range of lesion sizes commonly seen in clinical practice, with larger areas growing faster.^7–9^ To decrease test–retest variability and avoid the dependence of progression rate on baseline lesion size, Yehoshua et al.^10^ proposed taking the square root of the area to measure GA progression: the square root transformation (sqrt). This measured lineal (mm/year) rather than surface (mm^2^/year) progression and removed the correlation between BA and progression in many studies, which permitted to focus in other factors driving progression.^11–13^

However, as lesions grow they become more round-shaped, which may lead to a slowing of progression rate for very large lesions.^2,8,11,14,15^ Therefore, the relationship with progression rate may differ according to baseline lesion size even if measured with sqrt. This may be relevant for treatments where few patients with large lesions typically are included, such as stem-cell therapies. In these studies, it is common to use a paired design with the worst eye being selected systematically for the active group; if baseline size differs between eyes, an incorrect assumption about the treatment efficacy could be made.

We determined the relationship between BA and progression using the sqrt over a wide range of baseline sizes of atrophy. The aim was to characterize the applicability of sqrt in therapeutic and natural history studies of GA.

## Material and Methods

### Study Design

A retrospective review was conducted on data from patients who participated in the Characterization of Geographic Atrophy Progression in Patients With Age-Related Macular Degeneration (GAIN) study and others who would have been eligible for this study, but who visited at the Institut de la Màcula (Barcelona, Spain) after the end of this study. Briefly, the GAIN (NCT01694095)^9^ was a prospective natural history study of factors associated with GA progression conducted from December 2009 until August 2013; the medical history and imaging of these patients up to January 2018 were reviewed to increase their long term follow-up. To increase the sample size, we included new patients who met eligibility criteria who visited the clinic up to January 2018. The study followed the tenets of the Declaration of Helsinki and was approved by the Centro Médico Teknon ethics committee. An informed consent was obtained from all individual participants included in the study after explanation of the nature and possible consequences of the study.

### Inclusion and Exclusion Criteria

Men or women ≥50 years with GA (defined as a minimum diameter of complete RPE and outer retinal atrophy of 250 μm on spectral domain optical coherence tomography [SD-OCT])^16^ secondary to AMD and who had a follow-up ≥6 months were potentially eligible. This new definition of GA is less restrictive than that used previously (an area of RPE atrophy >0.5 disk areas [approximately 1.27 mm^2^]) and allowed the inclusion of more patients.

Exclusion criteria involved RPE atrophy deemed to be secondary to other causes (retinal dystrophy, high myopia, and so forth), previous neovascular AMD or other significant maculopathies, previous intraocular treatment (including laser photocoagulation, intravitreal injections or surgery, aside from phacoemulsification), inability to measure the whole area of atrophy, or insufficient image quality.

### Procedures

All patients underwent a complete ophthalmic exam as part of their regular visual care. This included medical history, best-corrected visual acuity (BCVA), intraocular pressure (IOP) and an anterior segment examination. After pupil dilatation with 1% tropicamide and 10% phenylephrine, posterior segment examination and fundus imaging were conducted. Imaging protocol included nontereoscopic 35° color fundus photography (Topcon TRC 50DX IA; Topcon, Tokyo, Japan); and 30° × 30° infrared (λ = 820 nm), fundus autofluorescence (FAF; excitation λ = 480 nm, emission λ between 500 and 700 nm), and 20° × 20°, 19 high-resolution (1536 × 1536 pixels) SD-OCT B-scans centered in the fovea with the Spectralis HRA+OCT (Heidelberg Engineering, Heidelberg, Germany) with a minimum automatic real time (averaging) of 10 images. Fluorescein angiography was performed only if required according to medical criteria.

The area of atrophy was measured on FAF by a single observer (MB) using the Region Finder software, versions 2.4.3.0 and 2.6.2.0 (Heidelberg Engineering), with good intraobserver agreement (see GAIN study).^9^ Rate of progression was determined by subtracting area of atrophy (in mm^2^) at the last visit from area of atrophy at baseline divided by time between visits (in years). For the sqrt measurements of progression (mm/year), the square root of the area on the last and first visits was calculated, subtracted, and the result was again divided by time between visits (in years).

### Statistical Analysis

Only one eye per patient was included, and in bilateral cases the study eye was randomly chosen. Univariate statistics were used to describe the sample, using means (± standard deviation [SD]) for quantitative variables and percentages for categorical variables, as appropriate.

The main outcome was the relationship between BA and GA progression using the sqrt (mm and mm/year, respectively) with Pearson *r* and Spearman *rho* correlation coefficients. This relationship also was plotted using linear regression and locally weighted scatterplot smoothing (LOWESS) curves with a tricube weighted function. These analyses were repeated in the original scale (mm^2^ and mm^2^/year) to check if the relationship was as expected.

As a secondary, prespecified analysis, progression on both metrics was compared by categories of BA (according to the Age-Related Eye Disease Study [AREDS] classification: 0.5 to <0.75, 0.75 to <4, and ≥4 DA)^11^ and by tertiles of BA using 1-way analysis of variance (ANOVA) tests. As a post hoc analysis, the relationship also was tested across subgroups defined by location of atrophy (foveal versus extrafoveal), number of lesions (unifocal versus multifocal), FAF pattern (“none” and “focal” versus “banded” and “diffuse”),^17^ and diagnosis of the fellow eye (drusen versus late AMD, GA or neovascular AMD).

Statistical analyses were conducted using Stata IC 15.1 (StataCorp, College Station, TX). A two-sided *P* value <0.05 was considered statistically significant.

## Results

We included 128 eyes from 128 patients with a mean follow-up of 3.1 (2.2) years. Mean age was 78.1 (7.9) years, 67.2% (86/128) were female, and all were Caucasian. Of the patients, 47.7% had bilateral GA and mean BCVA was 67.1 (17.0) letters, a Snellen equivalent of approximately 20/50. Mean baseline area of atrophy was 7.31 (6.55) mm^2^, mean sqrt at baseline was 2.44 (1.16) mm, and mean progression was 1.86 (1.12) mm^2^/year, with a sqrt of 0.33 (0.21) mm/year. Regarding lesion characteristics, 41.4% (53/128) had foveal atrophy; 67.2% (86/128) were multifocal; FAF pattern was none or focal in 29.7% (38/128), banded or diffuse in 67.2% (86/128), and other in 3.1% (4/128); and diagnosis of the fellow eye was drusen in 15.6% (20/128), late AMD in 80.5% (103/128), and other in 3.9% (5/128).

**Figure 1:**
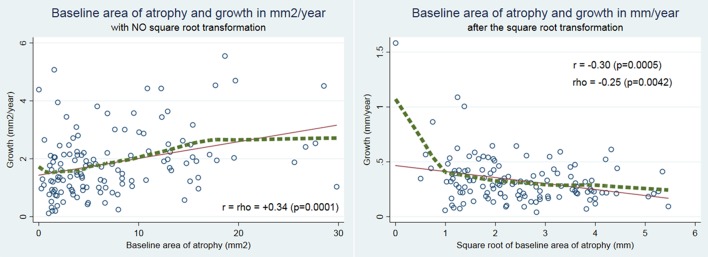
Left, relationship between progression and baseline area of atrophy on the conventional scales (mm^2^/year and mm^2^, respectively) in the whole sample. There is a positive correlation with larger baseline areas progressing faster, as expected. Right, relationship between progression and baseline area of atrophy after the square root transformation on both (mm/year and mm, respectively); there is a negative correlation. The LOWESS regression (green dotted line) does not differ markedly from the linear regression line (red solid line), except for the very few cases with small baseline areas of atrophy, <1 mm, after the transformation.

The main outcome, the correlation coefficient between BA and progression of GA in sqrt, was Pearson's *r* = −0.30, *P* = 0.0005 and Spearman's *rho* = −0.25, *P* = 0.0042. As expected, the correlation was positive for progression expressed in mm^2^/year, with Pearson's *r* and Spearman's *rho* = 0.34, *P* = 0.0001. These results are shown in Figure 1.

Secondary prespecified outcomes compared progression in both metrics, mm^2^/year and mm/year, using different classifications for BA (in mm^2^): AREDS and tertiles of the current sample. The results are shown in the Table, and were statistically significant for the sqrt tertiles of BA in our study (*P* = 0.0078), but not for the AREDS classification (*P* = 0.20). However, if the small lesion size category (0.5 to <0.75 DA) was increased to include lesions <0.5 DA (*n* = 27), then the progression in sqrt in this category became 0.44 (0.35) mm/year, and the comparison between categories reached statistical significance (*P* = 0.01). Therefore, the decreasing trend also was observed for BA in mm^2^. As expected, for measurements in mm^2^/year progression rate increased with increasing BA regardless of the classification used to stratify baseline lesion size (*P* ≤ 0.0002).

**Table:**
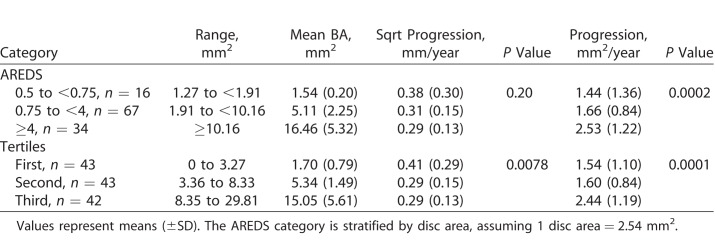
Comparison of Progression (in sqrt and in mm^2^/year) by Categories of Baseline Area of Atrophy

**Figure 2:**
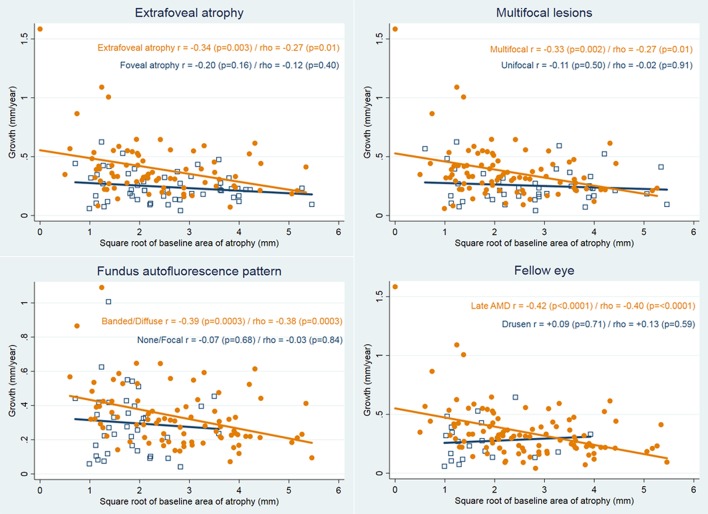
Relationship between growth and baseline area of atrophy using the square root transformation by subgroups. Top left, by foveal atrophy status. Top right, by number of lesions (uni/multifocal). Bottom left, by FAF pattern. Bottom right, by fellow eye status. The slopes are close to zero for conventional nonrisk characteristics, but they decrease markedly for features regarded as high-risk for progression (extrafoveal location of atrophy, multifocal lesions, patterns with high FAF and patients with late AMD in the fellow eye, either the neovascular form or geographic atrophy. Note that the “FAF figure” includes only the 124 cases classified as either none, focal, banded, or diffuse patterns (i.e., other patterns were excluded) and the “Fellow eye” figure is based on the 123 cases with fellow eye AMD.

Figure 2 shows the results across subgroups. A statistically significant negative slope was observed for the categories regarded as higher-risk for faster progression in each subgroup (eyes with extrafoveal atrophy, multifocal lesions, patterns characterized by high FAF and fellow eyes with late AMD), with *r* between −0.33 and −0.42 and *rho* between −0.27 and −0.40 (all *P* ≤ 0.01). This was not observed for lower-risk categories (foveal atrophy, unifocal lesions, patterns with no/minimally increased FAF, and fellow eyes with drusen), with *r* between +0.09 and −0.20 and *rho* between +0.13 and −0.12 (all *P* ≥ 0.16).

## Discussion

The results suggested that when using the sqrt method to track progression of GA, a decreasing progression rate is to be expected for increasingly large lesions. The results were robust for different analyses (correlation and mean comparison after categorization of BA), and were observed irrespective of the metric used to measure BA, either in mm (Fig. 1, right) or in mm^2^ (Table and Supplementary Figure S1).

A negative correlation coefficient between growth in mm/year and BA in mm was originally reported (Pearson's *r* = −0.09, Spearman's *rho* = −0.06),^10^ but this did not reach statistical significance (*P* > 0.40). This finding supported the assumption that the dependence on BA was eliminated with the sqrt. An increased magnitude of the correlation, a larger sample size, and/or a wider range of lesion sizes in our study may explain why some of our results were statistically significant, while others did not reach this conclusion.

The decreasing trend seen may be explained by the change in shape that occurs with lesion progression. Small lesions tend to be irregular and multifocal; when lesions become very large, they tend to coalesce and become more circular. This increase in circularity tends to decrease the perimeter-to-area ratio.^11,14,15^ A decrease in perimeter translates into less number of diseased RPE cells in contact with adjacent healthier cells, which may slow the lateral spread of the atrophy.^14^

A post hoc subgroup analysis was conducted to check if the results were similar across a range of known predictors of progression, namely atrophy location, number of lesions, FAF pattern and diagnosis of the fellow eye.^18^ As for the group overall, we found a moderate negative correlation in eyes with high-risk characteristics across the four groups, while eyes with low-risk characteristics showed a very low correlation. This suggested that growth in this metric slows down in very large lesion sizes in these particular subgroups. Small sample size in some categories with low-risk characteristics (i.e., in fellow eye diagnosis there were just 20 eyes with drusen) make results susceptible to a few outliers. Also, large lesions at baseline are required to observe this descending trend: we have shown that as lesions grow the linear progression (mm/year) decreases, probably because the growth is distributed along the whole perimeter of an increasingly large atrophic lesion. Figure 2 shows that the number of eyes with low-risk characteristics with a large BA square root (≥4 mm) is low (location of atrophy, multifocal lesions) or zero (FAF pattern, fellow eye), which may preclude the observation of this phenomenon. In fact, eyes may change from one category to another as BA enlarges. It is uncommon to see drusen only in the fellow eye of a patient with very large GA, but it is not rare to see them in the fellow eye of a patient with a small GA lesion. Patterns none and focal on FAF also are rarely observed in very large lesions.^17,19^ Given the exploratory nature of these analyses, they are regarded as hypothesis-generating and should be confirmed in further studies.

**Figure 3:**
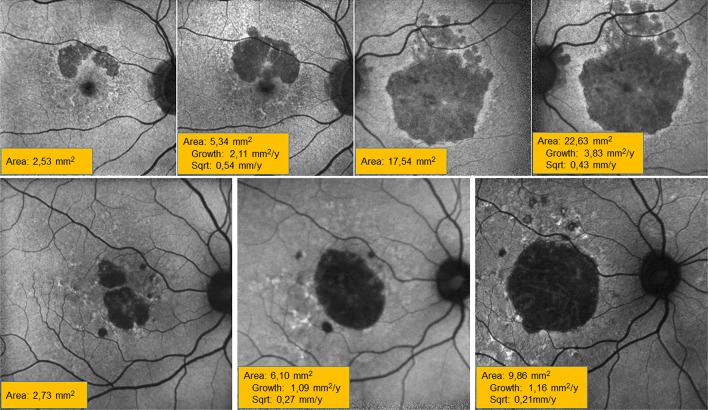
Comparison of progression rates between eyes of the same patient and different periods of time in the same eye in another subject. Top row, right (first two images) and left (last two images) eyes of the same patient after 16 months of follow-up. Progression rate is faster in mm^2^/year in the left eye, with a larger lesion, but linear progression in mm/year decreases as compared to the fellow eye. Bottom row, progression rate in mm^2^/year is larger in the second period (second to third image, 39 months) than in the first (first to second image, 37 months), but linear progression decreases with time by >20%.

The use of the sqrt is convenient to improve test–retest variability, to simplify trial design or to decrease the dependency on baseline lesion size.^10^ Nonetheless, it still may be reasonable to adjust for BA when evaluating risk factors for GA progression or when testing the efficacy of new therapies, especially in uncontrolled or small trials were randomization may not suffice to achieve a perfect balance between study arms in terms of important predictors. Otherwise, confounding may creep in. Special care should be taken in the context of advanced therapies, particularly stem cell treatments. In these studies, a paired design is not uncommon and the worst eye (usually that with the larger atrophy) may be selected systematically for treatment. If the sqrt is used, a slower progression is to be expected in the treated eye even in the absence of a real treatment effect, and, thus, claims of therapy efficacy may be mistakenly raised (Fig. 3).

The limitations of this study included a moderate sample size and a heterogeneous time of follow-up between patients. We only determined progression rate considering the first and last patient's visits, which may miss periods of acceleration and deceleration of lesion progression throughout the lesion lifespan, particularly for patients followed for a long period. The assumption of linear growth is particularly important in eyes with large lesions that eventually progress beyond the margins of FAF imaging, in which further enlargement is assumed to be of a similar rate as observed previously. Further research considering prospectively the baseline features and growth patterns across a range of BA is needed to isolate the predictive value of different factors on disease progression.

In summary, when GA progression is measured, a decrease in progression rate is to be expected for increasingly large lesions even using the sqrt method. While this does not preclude the use of this method, stratification or adjustment by BA of atrophy in prognostic or therapeutic studies on GA seems prudent.

## Supplementary Material

Supplement 1
