# Supplementary material for: The Rate of Progression of Geographic Atrophy Decreases With Increasing Baseline Lesion Size Even After the Square Root Transformation
Source: Transl Vis Sci Technol. 2018 Dec 28;7(6):40. doi: 10.1167/tvst.7.6.40 (PMC6314221; doi:10.1167/tvst.7.6.40)
Supplement: Supplement 1 [file tvst-07-06-30_s01.pdf]

SUPPLEMENTARY MATERIAL

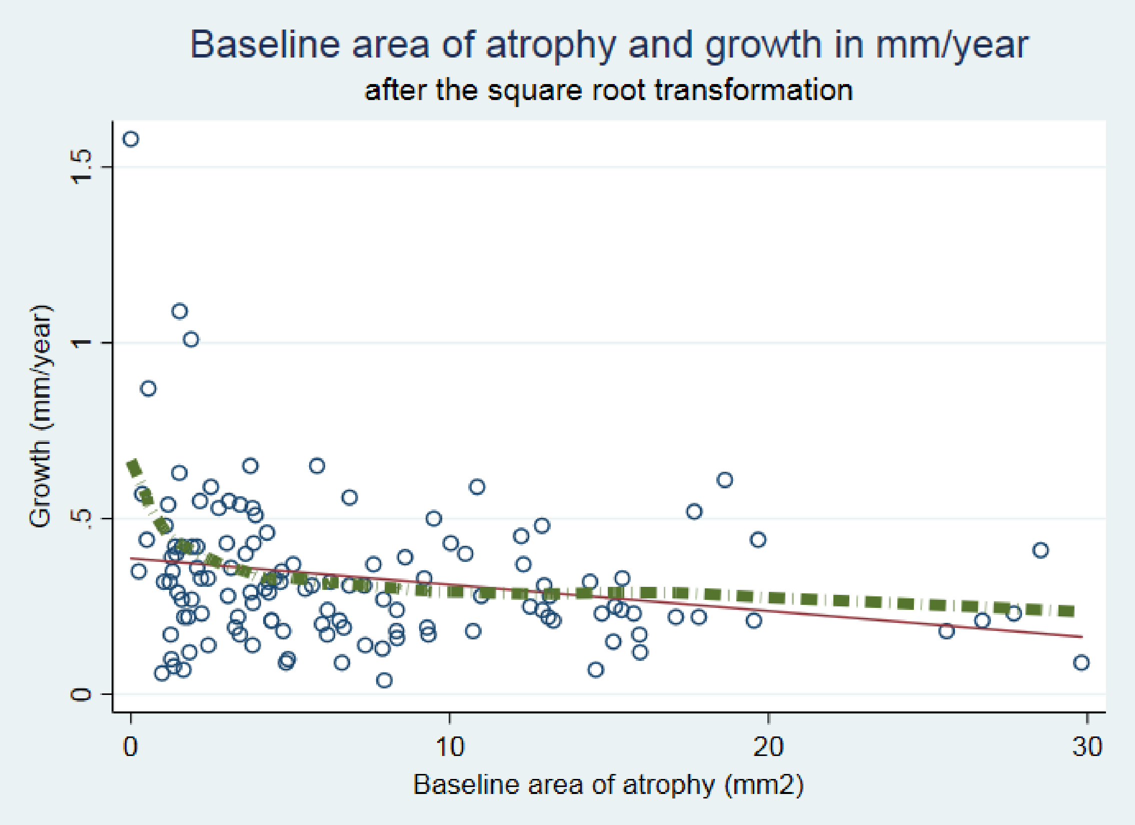

Supplementary Material Figure 1. Relationship between growth (after the square root transformation) and baseline area of atrophy *(in mm^2)*. The correlation is also negative, with Pearson's *r* = -0.23 (p=0.0087) and Spearman's *rho* = -0.25 (p=0.0042), with a more marked negative slope for smaller lesions.
